# The Effects of Short-Term Exposure to Low Temperatures During the Booting Stage on Starch Synthesis and Yields in Wheat Grain

**DOI:** 10.3389/fpls.2021.684784

**Published:** 2021-07-09

**Authors:** Wenjing Zhang, Yan Zhao, Lingyu Li, Xu Xu, Li Yang, Zheng Luo, Beibei Wang, Shangyu Ma, Yonghui Fan, Zhenglai Huang

**Affiliations:** ^1^Key Laboratory of Wheat Biology and Genetic Improvement on South Yellow and Huai River Valley, The Ministry of Agriculture, Hefei, China; ^2^Department of Agronomy, Anhui Agricultural University, Hefei, China

**Keywords:** wheat, low temperature at booting, starch synthesis enzymes, starch content, grain-filling, yield

## Abstract

Low temperatures (LT) in spring can have a major impact on the yields of wheat in winter. Wheat varieties with different cold sensitivities (the cold-tolerant Yannong 19 variety and the cold-sensitive Yangmai 18 variety) were used to study the responses of the wheat grain starch synthesis and dry material accumulation to short-term LT during the booting stage. The effects of short-term LT on the activities of key wheat grain starch synthesis enzymes, starch content and grain dry-matter accumulation were determined by exposing the wheat to simulated LT of from −2 to 2°C. Short-term LT stress caused a decrease in the fullness of the wheat grains along with decreased activities of adenosine diphosphate glucose pyrophosphorylase (AGPase, EC2.7.7.27), soluble starch synthase (SSS, EC2.4.1.21), granule-bound starch synthase (GBSS, EC2.4.1.21), and starch branching enzyme (SBE, EC2.4.1.18) at different spike positions during the filling stage. The rate of grain starch accumulation and starch content decreased with decreasing temperatures. Also, the duration of grain filling increased, the mean and the maximum filling rates were reduced and the quality of the grain dry-matter decreased. The number of grains per spike and the thousand-grain weight of the mature grains also decreased. Our data showed that short-term LT stress at the booting stage caused a decrease in the activities of key starch synthesis enzymes at the grain-filling stage. These changes reduced the accumulation of starch, decreased the filling rate, and lowered the accumulation of grain dry matter to ultimately decrease grain yields.

## Introduction

Low temperature stress is a major limiting factor seriously affecting global wheat production and development (Xiao et al., 2021). During springtime, the wheat-producing regions of Huang-Huai-Hai and the middle and lower reaches of the Yangtze River in China experience low temperatures (LT) that can act as major abiotic stresses and limit wheat production later in the season (Fang et al., 2011). LT in spring mostly occurs during the jointing to booting stages of wheat development. This can have major impacts on the morphology and growth resulting in decreased spike numbers, grain numbers and the thousand-grain weight (Zheng et al., 2015). Overall, low temperature has severely affected the production of wheat in the world (Holman et al., 2011; Zhang et al., 2011; Crimp et al., 2016).

Starch is the most important carbohydrate in wheat grains that accounts for 63–70% of the grain weight. The dynamics of starch contents and the accumulation of starch determines the yield and quality of wheat. Starch synthesis is mainly synthesized in the endosperm cells and regulated by a series of enzymes (Ran et al., 2020). Adenosine diphosphate glucose pyrophosphorylase (AGPase, EC2.7.7.27), soluble starch synthase (SSS, EC 2.4.1.21), granule-bound starch synthase (GBSS, EC2.4.1.21), and starch branching enzyme (SBE, EC2.4.1.18) have particularly important roles in starch synthesis (Wang et al., 2021). AGPase catalyzes the reaction between glucose-1-phosphate (G-1-P) and ATP to form adenosine diphosphate glucose (ADPG) and pyrophosphoric acid (PPi). This is a rate-limiting step in endosperm starch synthesis that is directly determined by the rate of starch synthesis and accumulation (Bowsher et al., 2007). SSS catalyzes the synthesis of amylopectin (Zhang et al., 2010) and a high level of SSS activity is required to improve the level of starch synthesis (Zhang G. Y. et al., 2011). AGPase and SSS enzymes are key enzymes in regulating rice starch synthesis (Prathap and Tyagi, 2020). GBSS participates in amylose synthesis by specifically binding to starch granules. SBE participates in the formation of α-1,6 glycoside branched-chain to synthesize amylopectin. In cereal endosperm, the deficiency of SBE could change the morphology and structure of starch to form heterogeneous starch granules (He and Wei, 2020).

The rate of accumulation of straight and branched-chain starch, and the total starch in wheat grains is directly correlated with the activities of SBE, SSS, GBSS, and AGPase (Wang et al., 2014). The expression of genes and the activities of enzymes related to starch synthesis in grains are sensitive to temperature and water stress (He et al., 2012; Cao et al., 2015). AGPase, SSS, and SBE activities decrease significantly at mid-late grain filling stage by water deficit (Mahla et al., 2017). Hurkman et al. (2003) found that high temperatures between anthesis and maturity increase the peak of enzyme activity relative to starch synthesis and reduce enzyme activity to shorten the duration of starch accumulation. LT during the filling stage significantly reduces the starch content in bread wheat grains (Labuschagne et al., 2009).

To date, most studies have focused on the effects of LT in spring on the growth, development and yields of wheat. Previous studies have reported significantly decreased rates of photosynthesis in functional wheat leaves caused by LT at the booting stage (Zhang W. J. et al., 2015). Also, LT can decrease carbohydrate accumulation (Zeng et al., 2011) leading to changes in the content of endogenous hormones and sucrose metabolism in young spikes (Zhang W. J. et al., 2019). However, few reports have characterized the effects of short-term LT at the booting stage especially the connective stage on starch synthesis in wheat grains.

This study aimed to analyze changes in the activities of enzymes associated with starch synthesis, starch accumulation, dry matter accumulation, and wheat yields after short-term LT stress at the booting stage in wheat varieties with different cold sensitivities. Also, we aimed to reveal the effects of short-term LT at this stage on grain development.

## Materials and Methods

### Plant Materials

Two wheat varieties with differing sensitivities to LT were selected, the tolerant variety Yannong 19 (bred by Shandong Yantai Academy of Agricultural Sciences) and the sensitive variety Yangmai 18 (bred by Jiangsu Lixiahe Academy of Agricultural Sciences).

### Experimental Design

**Figure 1:**
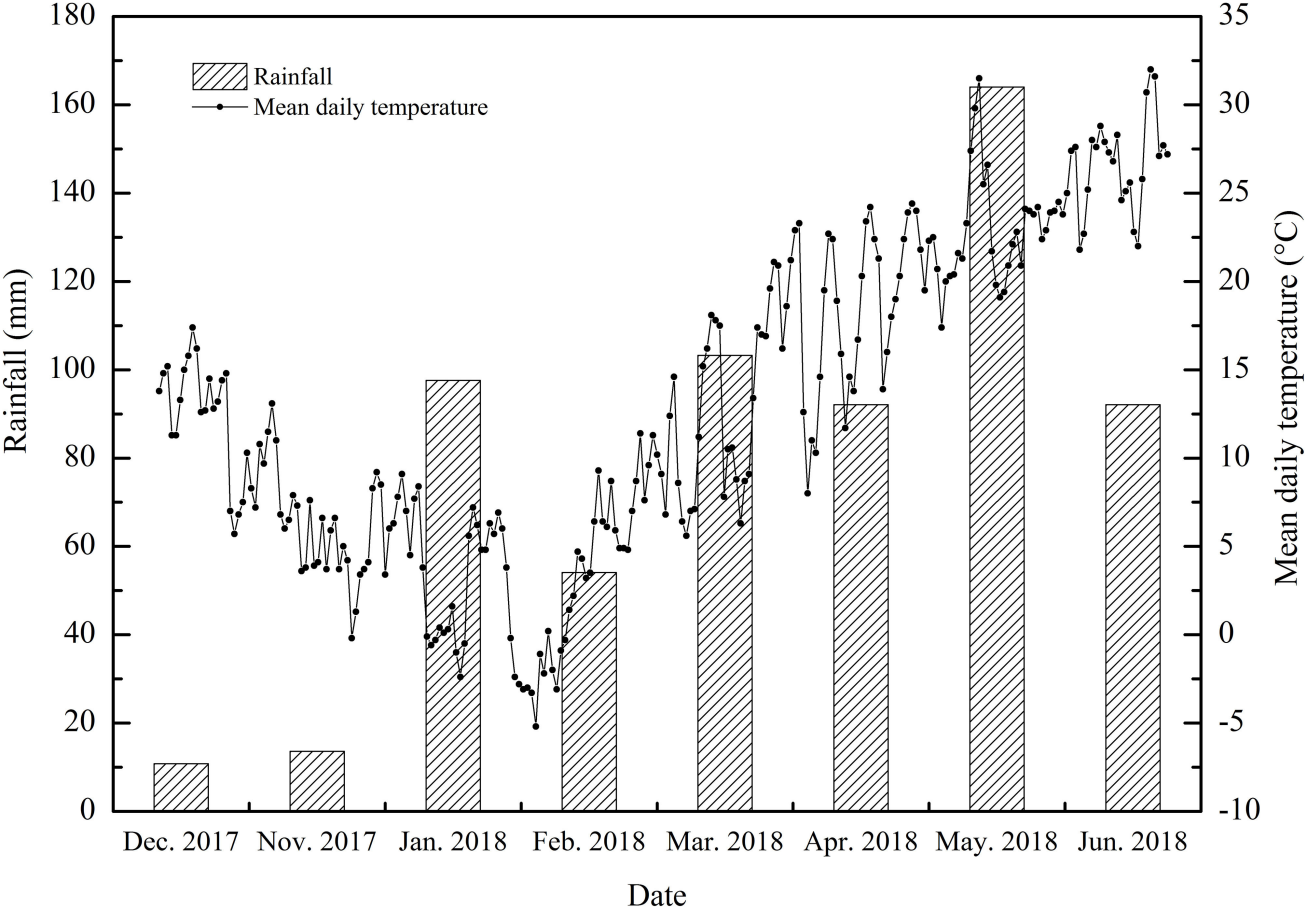
The average daily temperatures and monthly rainfall during the wheat-growing seasons (2017–2018).

The experiments were carried out at the experimental base of the Anhui Agricultural University (Hefei, Anhui) from November 2017 to June 2018 (31.52°N, 117.17°E). The subtropical humid monsoon climate zone was selected as the test site. The mean annual temperature was between 15 and 16°C, the mean annual precipitation was 900–1,000 mm, the annual sunshine total was 2,000 h and the mean annual frost-free period was 228 d. The meteorological data of the wheat growing season in this study including the mean daily temperature and the mean monthly rainfall are summarized in Figure 1.

The experiment used a combination of potted and field planting methods. The pots were 30 cm high and 30 cm in diameter. The potting soil was taken from a 0 to 20 cm cultivation layer. The soil organic matter content was 14.80 g·kg^−1^, the total nitrogen was 0.81 g·kg^−1^ and the potassium, nitrogen, phosphorus, and concentrations were 102.4, 23.6, and 152.7 mg·kg^−1^, respectively. Each pot was filled with 10 kg of sieved soil. The plants were sown on November 2nd, 2018. Before sowing, 75 g of organic fertilizer, 2.26 g of pure nitrogen, and 7 g of compound fertilizer were added to each pot followed by the addition of 1.05 g of pure nitrogen at the jointing stage. For each variety, 120 pots were planted and subsequently buried in the experimental field so that the upper edge of the pots was flush with the ground. The seedlings were thinned so that 10 seedlings were left in each pot. All other field management measures were performed according to the requirements of high-yield cultivation.

Wheat is sensitive to low temperature between the pistil and stamen differentiation stage and the connective stage. Based on the time of the occurrence of LT in spring and observed the stage of spike differentiation with a microscope, 90 pots of each variety were placed in an artificial climate chamber (ACC) for LT treatment on April 3rd, 2018. The plants were housed at temperatures of −2, 0 or 2°C from 19:00 to 07:00, and from 07:00 to 19:00, the temperature was set to 5°C. The processing time was 24 h and the atmospheric humidity in the climate chamber was set to 70%. At the end of the LT treatment, the pots were transferred back to the field to allow the plants to mature.

### Sample Harvesting

After anthesis, the wheat ears were marked to indicate the same period of growth and the flowering date. From 10 days after anthesis, 15–20 equally sized wheat ears were selected every 5 days. Five of the ears were washed, quickly frozen in liquid nitrogen and stored at −80°C to determine the activity of enzymes related to starch synthesis. The remaining grains were removed from the wheat ears and fixed at 105°C for 15 min. The grains were then dried at 80°C to constant weight and the values were used to determine the starch content and accumulation of grain dry matter. The two wheat varieties tested generally contained 18–20 spikelets per ear. The central six spikelets were termed the middle spikelets, the upper six to seven spikelets were termed upper spikelets, and the basal six to seven spikelets were termed the lower spikelets.

### Measurements of Grain Parameters

#### Grain Morphology

From 10 days after anthesis, fresh wheat grains from the LT treatment and control were sampled every 5 days. For each sample, 1–2 grains at the base of the middle spikelet were harvested and observed using a SZX16 stereomicroscope (OLYMPUS, Japan).

#### The Activity of Enzymes Related to Starch Synthesis

10–15 grains were sampled and weighed. Five mL of 100 mmol.L^−1^ Tricine-NaOH extract [(pH 8.0, 10 mmol·L^−1^ MgCl_2_, 2 mmol·L^−1^ EDTA, 50 mmol·L^−1^ 2-mercaptoethanol, 12% (v/v) glycerol, 5% (w/v) insoluble polyvinylpyrrolidone-40] was added to the samples that were then ground in a mortar at 0°C and centrifuged at 10,000 × *g* for 10 min (4°C). The supernatants were used to determine the activities of AGPase, SSS, and SBE. The samples were centrifuged and the pellets washed once with 800 μL 100 mmol·L^−1^ Tricine-NaOH extract. The pellets were then suspended in 100 mmol·L^−1^ Tricine-NaOH extract to give a crude enzyme solution that was used to determine the GBSS enzyme activity.

The activity of AGPase activity was determined following the method of Nakamura et al. (1989). 20 μL crude enzyme extract was added to 110 μL reaction solution [100 mmol·L^−1^ Hepes-NaOH (pH 7.4), 1.2 mmol·L^−1^ ADPG, 3 mmol·L^−1^ PPi; 5 mmol·L^−1^ MgCl_2_, 4 mmol·L^−1^ DTT]. The mixture was incubated at 30°C for 20 min and the reaction was terminated by placing in boiling water for 30 s. The solution was centrifuged at 10,000 × *g* for 10 min, then 100 μL supernatant was added to 5.2 μL colorimetric solution (5.76 mmol·L^−1^ NADP, 0.08 unit P-gucomutase, 0.07 unit G-6-P dehydrogenase). After incubation at 30°C for 10 min, the OD value at 340 nm was measured.

The activities of SSS and GBSS were measured as previously described by Nakamura et al. (1989) and Jiang et al. (2003). Crude enzyme extract (20 μL) was added to 36 μL of the reaction solution [(50 mmol·L^−1^ HEPES-NaOH (pH 7.4), 1.6 mmol·L^−1^ ADPG, 0.7 mg amylopectin, 15 mmol·L^−1^ DTT)] and incubated at 30°C for 20 min. The reaction was stopped by heating in boiling water for 30 s followed by cooling in an ice bath. 20 μL of reaction solution [(containing 50 mmol.L^−1^ HEPES-NaOH (pH 7.4), 4 mmol.L^−1^ PEP, 200 mmol.L^−1^ KCl, 10 mmol·L^−1^ MgCl_2_, 1.2 units pyruvate kinase)] was added and the samples incubated at 30°C for 20 min. The reaction was stopped by heating in boiling water. The samples were centrifuged at 10,000 × *g* for 10 min and 60 μL of supernatant was added to 40 μL of the reaction solution [50 mmol·L^−1^ HEPES-NaOH (pH 7.4), 10 mmol·L^−1^ glucose, 20 mmol·L^−1^ MgCl_2_, 2 mmol·L^−1^ NADP, 1.4 units hexokinase, 0.35 units G-6-P dehydrogenase)]. The mixture was incubated at 30°C for 10 min and the OD measured at 340 nm.

The activity of SBE was determined according to the methods of Nakamura et al. (1989) and Yang et al. (2004). Crude enzyme solution (20 μL) was added to 20 μL reaction solution [final concentration 50 mmol·L^−1^ HEPES-NaOH (pH 7.4), 5 mmol·L^−1^ G-l-P, 1.25 mmol·L^−1^ AMP, phosphorylase (5.4 units)]. After incubation of the reaction for 30 min at 30°C, 10 μL 1 mol·L^−1^ HCl was used to terminate the reaction, and 100 μL dimethylsulfoxide and 140 μL of 0.1% I_2_ and 1% KI were added and the OD value at 540 nm was determined.

#### Starch Content

The wheat grains were dried, ground into a powder and passed through a 100-mesh sieve. Eight mL of 80% ethanol was added to 0.1 g of each sample. The samples were then heated in a water bath at 80°C for 30 min, allowed to cool and centrifuged at 5,000 × *g* for 15 min. The supernatant was discarded and the precipitate was dried at 60°C. Distilled water (2 mL) was added to the precipitate, the solution was boiled for 20 min and then allowed to cool before adding 2 mL of 9.2 mol·L^−1^ HClO_4_. The samples were shaken for 10 min. Six mL of distilled water was added to each sample before being centrifuged at 5,000 × *g* for 15 min. The supernatant was decanted into a 50-mL volumetric flask. This process was repeated three times and the volume of each sample was adjusted to a constant volume. 0.1 mL of extract was then added to 4 mL of 0.2% anthrone. The samples were boiled for 15 min and the OD measured at 620 nm.

The logistic equation *y* = W/[1 + e^(A+Bt)^] (Darroch and Baker, 1990) was used to relate the variations in starch content (y) to the number of days after anthesis (t), where W is the final theoretical starch content, and A and B are constants. By calculating the first derivative of the equation, we obtained the following formula:

(1) $$\begin{array}{l} \text{Starch accumulation rate}\left(V_{\text{t}}\right):V_{\text{t}}=\text{-WC}{\text{e}}^{\text{(A+Bt)}}\text{/(1+}{\text{e}}^{\text{(A+Bt)}}{\text{)}}^{\text{2}} \end{array}$$

#### Grain Dry-Matter Accumulation

After anthesis, wheat ears that had bloomed on the same day and had developed to the same size were marked. From 10 days after anthesis, 8–10 wheat ears with uniform growth were selected every 5 days. The grains were removed and the seeds were fixed at 105°C for 15 min. The grains were dried at 80°C to a constant weight. The samples were weighed and converted into a thousand-grain weight. The logistic equation *Y* = K/[1 + e^(a+bt)^] was used to associate the variations in grain weight (Y) with the number of days after anthesis (t), where K is the fitted maximum grain weight, and a and b are constants (Darroch and Baker, 1990). The first and second derivations of the equation were used to derive:

(2) $$\begin{array}{l} \text{The duration of incremental filling period }\left(T_{1}\right):\\ T_{1}=(\text{a}-1.317)/\text{b} \end{array}$$

(3) $$\begin{array}{l} \text{The duration of rapid filling period }\left(T_{2}\right):\\ T_{2}=(\text{a}+1.317)/\text{b}-(\text{a}-1.317)/\text{b} \end{array}$$

(4) $$\begin{array}{l} \text{The duration of slow filling period }\left(T_{3}\right):\\ T_{3}=T-T_{1}-T_{2} \end{array}$$

(5) $$\begin{array}{l} \text{The time when the maximum grain filling rate appears }\left(T_{\text{max}}\right):\\ T_{\text{max}}\;=\;-\text{a}/\text{b} \end{array}$$

(6) $$\begin{array}{l} \text{Grain filling duration }\left(T\right):\\ T\;=\;\;\left[\text{ln}(1/9-\text{a})\right]/\text{b} \end{array}$$

(7) $$\begin{array}{l} \text{Mean filling rate }\left(R\right):R\;=\text{K}/T \end{array}$$

(8) $$\begin{array}{l} \text{Maximum filling rate }\left(R_{\text{max}}\right):\\ R_{\text{max}}\;=\;-\text{K}*\text{b}/4 \end{array}$$

#### The Number of Grains per Spike and Thousand-Grain Weight

After the wheat had matured, the ears from 20 pots that had not been sampled were randomly selected to calculate the number of grains per spike and the thousand-grain weight. The measurements were repeated three times.

### Data Analysis

SPSS 10.0 software (version 10; IBM, Corp., Armonk, NY, USA) was used to analyze the variance of the data. Data were subjected to analysis of variance (ANOVA), and Duncan's method was used for multiple comparisons among treatments (*P* < 0.05). Curve fitting of starch content and grain dry-matter data were conducted with IBM SPSS Statistics 20 (version 20; IBM, Corp., Armonk, NY, USA) and relevant parameters were calculated. The Pearson's correlation coefficient was used to calculate the coefficients and *p*-values. Origin 8.0 was used to generate the graphical representations of the data.

## Results

### The Effect of LT at the Booting Stage on Grain Morphology

**Figure 2:**
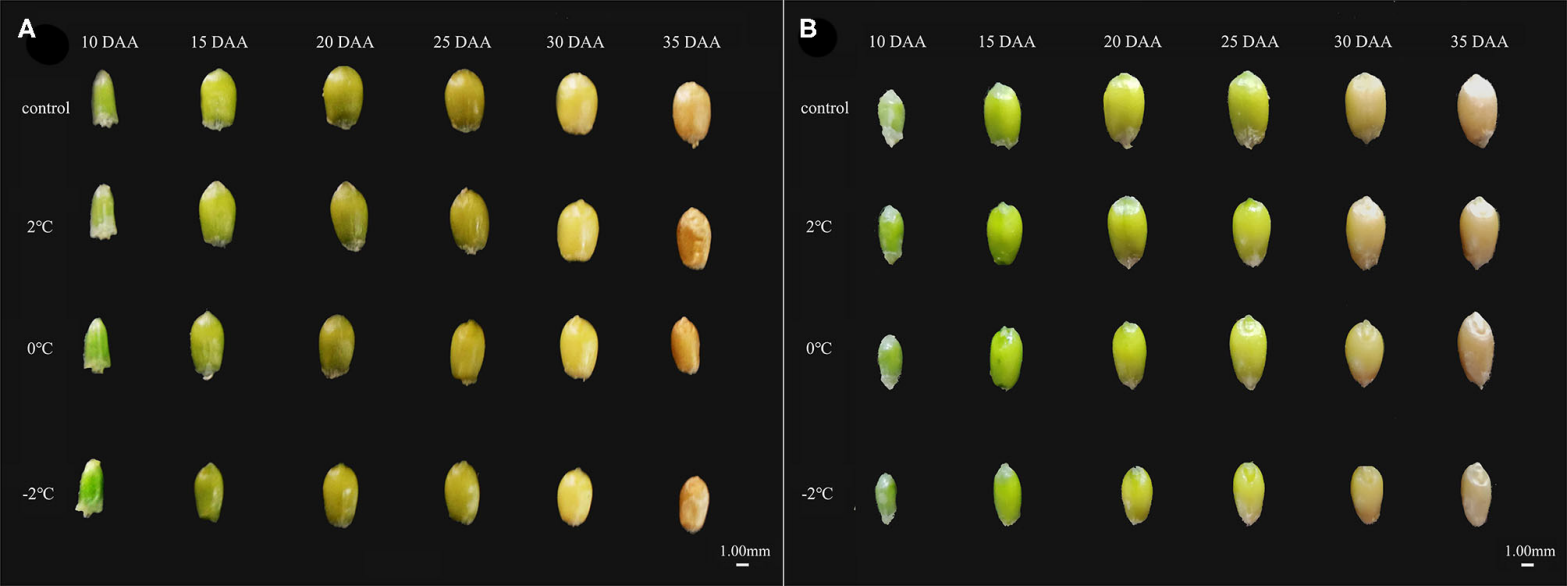
The effects of short-term low temperature (LT) at the booting stage on the morphology of grains during the grain filling stage (DAA, day after anthesis). At the booting stage, the sensitive (Yangmai 18, **A**) and the tolerant (Yannong 19, **B**) varieties were placed in the artificial climate chamber for LT stress. The temperature was set to 5°C during 07:00–19:00 and at 2, 0, and −2°C during 19:00–07:00. The normal growing plants without cold stress were used as controls.

Short-term LT stress at the booting stage affected wheat grain filling (Figure 2). The fullness of the grains after LT stress was poor compared to the grains that were not subjected to LT stress at the booting stage. The grain volume during the entire filling stage was lower than that of the controls. Also, the grain size decreased concomitantly with a decrease in the temperature indicating that short-term LT stress at the booting stage affects the grain development.

### The Effects of LT at the Booting Stage on the Activities of Starch Biosynthesis Enzymes in Grains

#### APGase Activity

**Figure 3:**
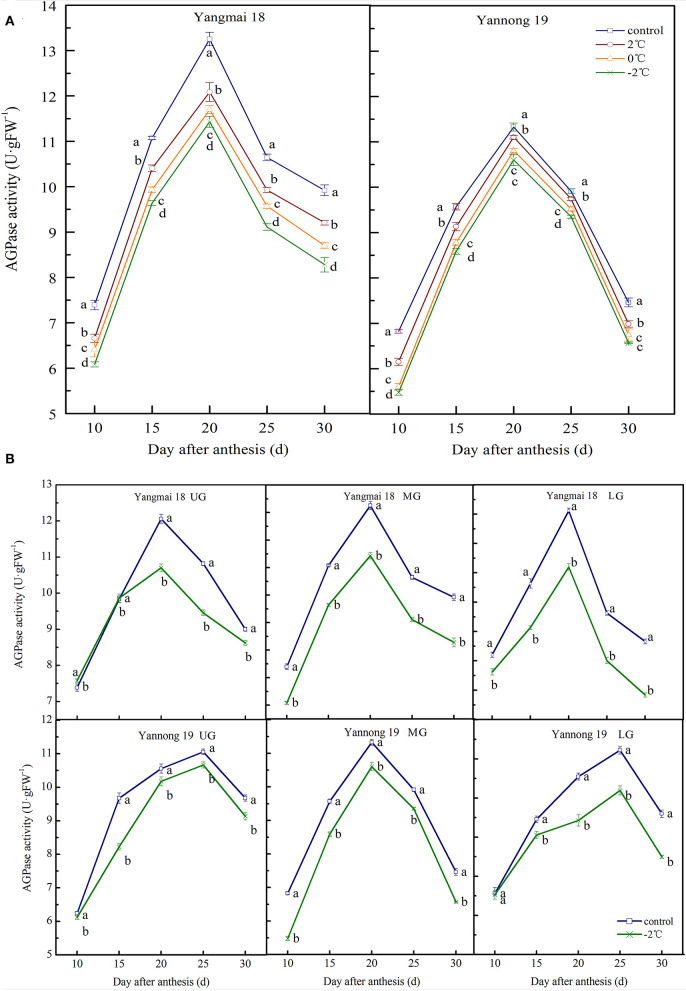
The effect of short-term LT at the booting stage on the ADP-glucose pyrophosphorylase activity of wheat grains in the middle spikelets **(A)** and at different positions of the spikelets **(B)**. UG, grains in the upper spikelets; MG, grains in the middle spikelets; LG, grains in lower spikelets. At booting stage, wheat plants were placed in the artificial climate chamber for LT stress. The temperature was set to 5°C during 07:00–19:00 and at 2, 0, and −2°C during 19:00–07:00. The normal growing plants without cold stress were used as controls. The data are presented as the means ± SD (*n* = 3). Different lowercase letters indicate significant differences between the treatments determined using the Duncan's multiple range test (*P* < 0.05).

The activity of AGPase in wheat grains at the filling stage showed a single-peak curve. After short-term LT stress at the booting stage, the enzyme activity was significantly decreased (Figures 3A,B) compared to the activity in the control group (*P* < 0.05). For the middle spikelet, the AGPase activity in the Yannong 19 and Yangmai 18 grains reached a peak around 20 days after anthesis and then rapidly declined (Figure 3A). After a short period of LT (2, 0 or −2°C) treatment at the booting stage, the AGPase activity was lower than in the control group during the entire filling stage. For example, at 20 days after anthesis, LT (2, 0, and −2°C) decreased AGPase activity in the grains of the middle spikelet of Yangmai 18 by 8.81, 11.75, and 16.53%, respectively, compared to the activities in the control group. In the Yannong 19 variety, the activity of AGPase decreased by 1.99, 4.60, and 6.40% at temperatures of 2, 0, and −2°C, respectively, compared to the control group.

The AGPase activity in grains at different spike positions decreased after −2°C treatment (Figure 3B). The AGPase activity in the upper, middle and lower spikelets of the Yangmai 18 variety reached a peak at around 20 days after anthesis. The peak enzyme activities decreased by 11.25, 13.77, and 15.83%, respectively, compared to the controls. In the Yannong 19 variety, the AGPase activity of the middle spikelet grains peaked at 20 days after anthesis, whilst for the upper and lower spikelet grains, the activity peaked at 25 days after anthesis. The timings of the peaks in the LT treatment groups were also consistent with those in the control group. The peak enzyme activities of the upper, middle and lower spikelets of the Yannong 19 variety decreased by 3.53, 6.40, and 10.06%, respectively, compared to the control spikelets. The AGPase activity in the LT-sensitive Yangmai 18 variety was more sensitive to variations in temperature compared to the Yannong 19 variety.

#### SSS Activity

**Figure 4:**
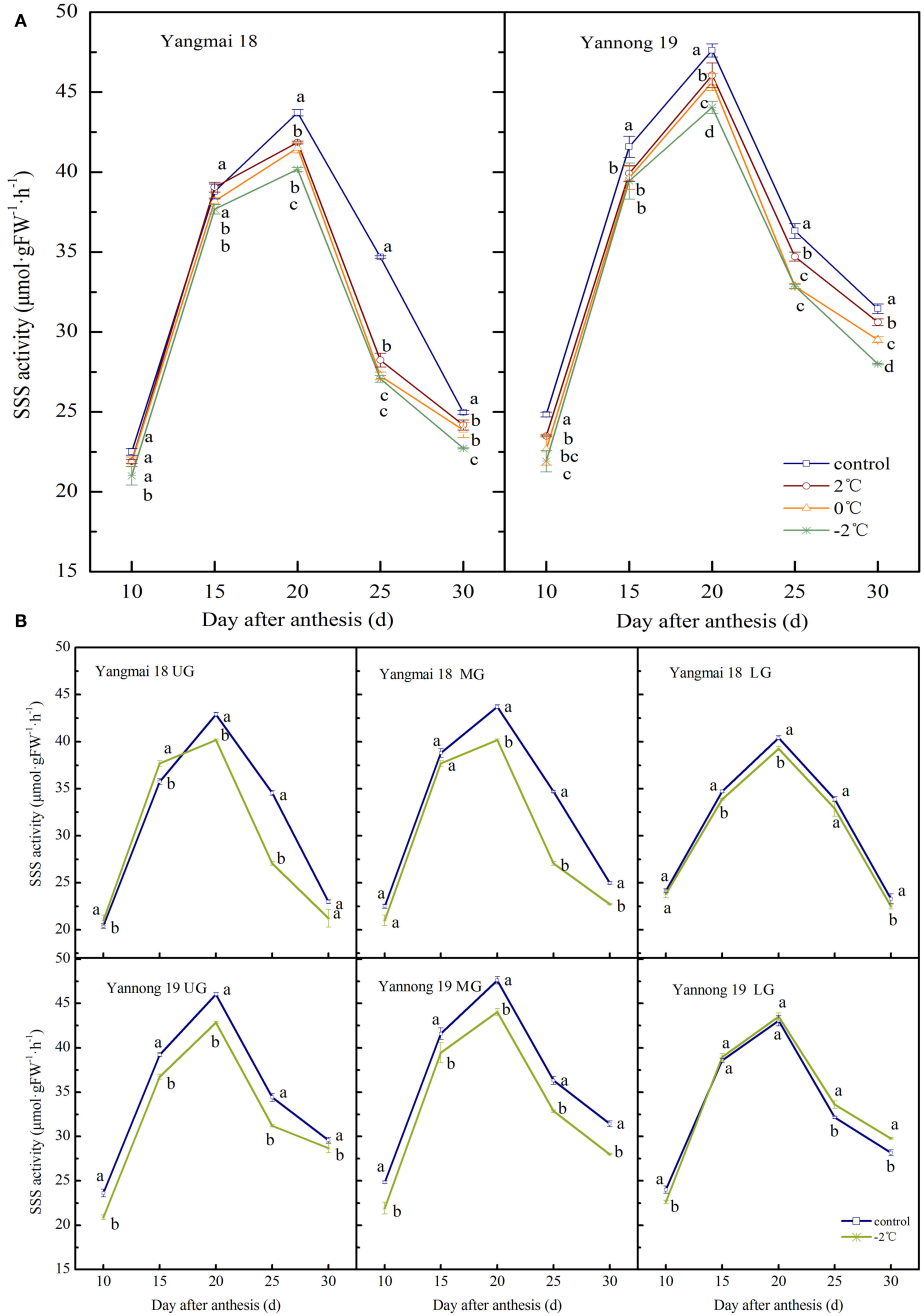
The effect of short-term LT at the booting stage on the activity of soluble starch synthase in the wheat grain of the middle spikelets **(A)** and different position spikelets **(B)**. UG, grains in the upper spikelets, MG, grains in the middle spikelets, LG, grains in the lower spikelets. At the booting stage, the wheat plants were placed in an artificial climate chamber for LT stress. The temperature was set to 5°C during 07:00–19:00 and 2, 0, and −2°C during 19:00–07:00. The normal growing plants without cold stress were used as controls. The data are presented as the means ± SD (*n* = 3). Different lowercase letters indicate significant differences between the treatments determined using the Duncan's multiple range test (*P* < 0.05).

The activity of SSS in wheat grains at the grain-filling stage showed a single-peak curve and the peak enzyme activity appeared around 20 days after anthesis (Figures 4A,B). After exposure to short-term LT stress at the booting stage, the SSS activity decreased (except for in the lower spikelet of Yannong 19). The activity of SSS in the middle spikelet grains increased consistently with decreasing treatment temperature (Figure 4A). Significant differences in the activity of SSS were observed in grains in the LT treatment and control groups (*P* < 0.05, except for Yangmai 18 at 10 d and 15 d after anthesis). At 20 days after anthesis, the activity of SSS in the grains of the middle spikelet of the Yangmai 18 variety decreased by 4.27%, 5.05% and 8.12% after treatments at 2, 0, and −2°C, respectively, and in the Yannong 19 variety, the corresponding SSS activities decreased by 3.27, 4.15, and 7.48%.

For grains at different spike positions (except for the lower spikelets of Yannong 19), the SSS activity decreased after treatment at −2°C. The peak SSS activity for grains in the upper, middle and lower spikelets of the Yangmai 18 variety decreased by 6.38, 8.12, and 2.84%, respectively, compared to the grains at the respective positions in the control group. The peak SSS activity in the upper and middle grains of the Yannong 19 variety decreased by 6.91 and 7.48%, respectively, compared to the grains in the control. The activities of SSS in the lower grains increased slightly compared to the control (1.10%). A comparison between the varieties showed that the SSS activity in Yangmai 18 grains decreased after LT stress, however, the observed changes in the SSS activity of Yannong 19 grains were irregular.

#### GBSS Activity

**Figure 5:**
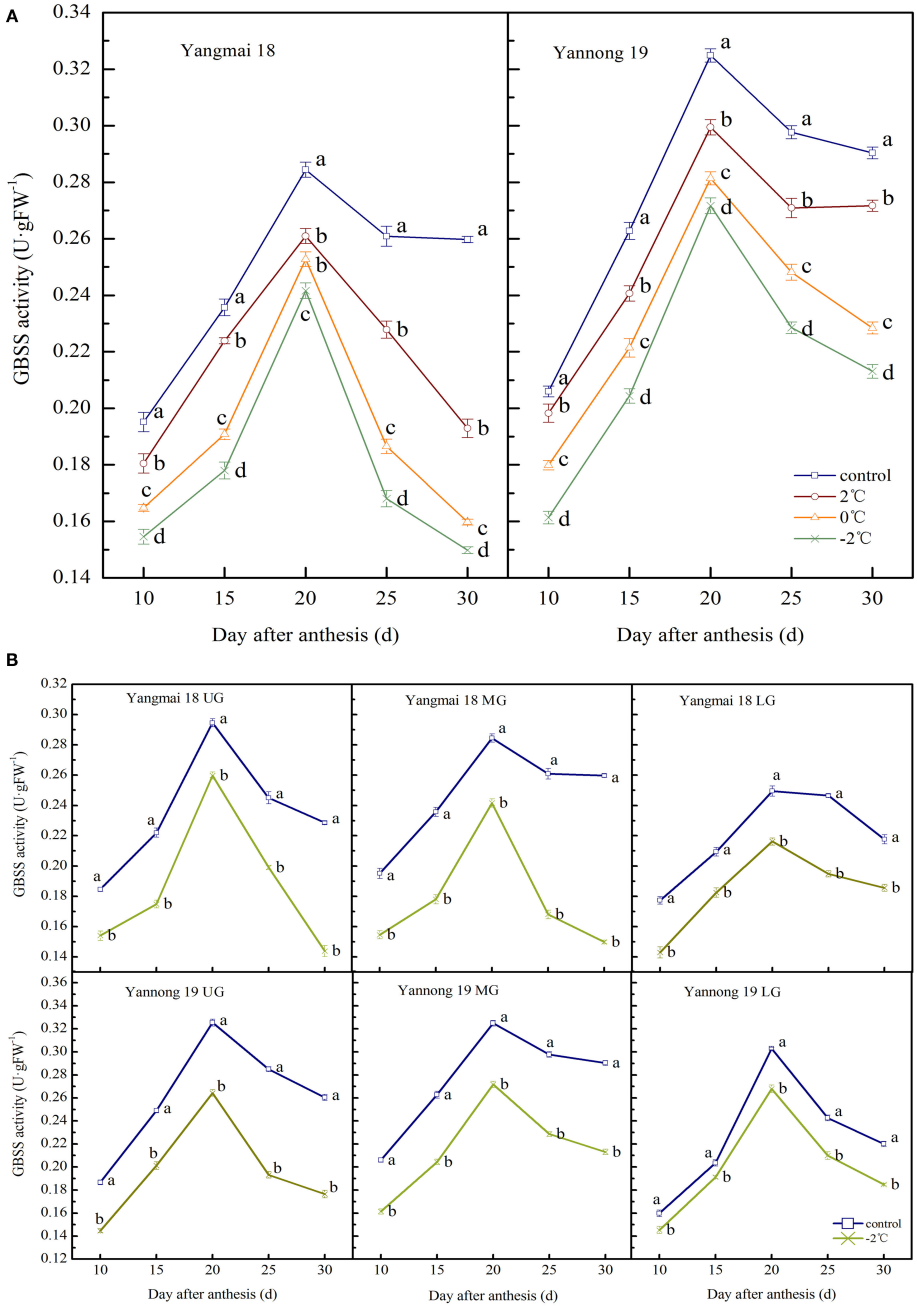
The effect of short-term LT at the booting stage on the granule-bound starch synthase activity of wheat grain in the middle spikelets **(A)** and different position spikelets **(B)**. UG, grains in the upper spikelets; MG, grains in the middle spikelets; LG, grains in the lower spikelets. At the booting stage, the wheat plants were placed in an artificial climate chamber for LT stress. The temperature was set to 5°C during 07:00–19:00 and 2, 0, and −2°C during 19:00–07:00. The normal growing plants without cold stress were used as controls. The data are presented as the means ± SD (*n* = 3). Different lowercase letters indicate significant differences between the treatments determined using the Duncan's multiple range test (*P* < 0.05).

The enzyme activity of GBSS in wheat grains at the filling stage showed a single-peak curve that reached peak activity at around 20 days after anthesis (Figures 5A,B). After a short period of LT (2, 0, and −2°C) treatment at the booting stage, the activity of GBSS in the grains of the two varieties was significantly lower than the control group during the entire filling period (*P* < 0.05). At 20 days after anthesis, after treatment at 2, 0, and −2°C, the GBSS enzyme activity in the grains of the middle spikelet of the Yangmai 18 variety decreased by 8.26, 11.14, and 15.05%, respectively. In the Yannong 19 variety, corresponding decreases of 7.83, 13.40, and 16.38%, were observed compared to the control group.

The GBSS activities of grains at different spike positions after treatment at −2°C were significantly lower than the control group during the whole filling period (*P* < 0.05, Figure 5B). The GBSS activity peak for grains in the upper, middle and lower spikelets of the Yangmai 18 variety decreased by 11.81, 15.05, and 13.35%, respectively, compared to the corresponding controls. A similar decreasing trend was observed in the Yannong 19 variety with corresponding decreases in GBSS activities of 18.79, 16.38, and 11.56%, compared with the controls. These data indicate that the GBSS activity of Yannong 19 grains was higher than the Yangmai 18 grains during the whole filling period.

#### SBE Activity

**Figure 6:**
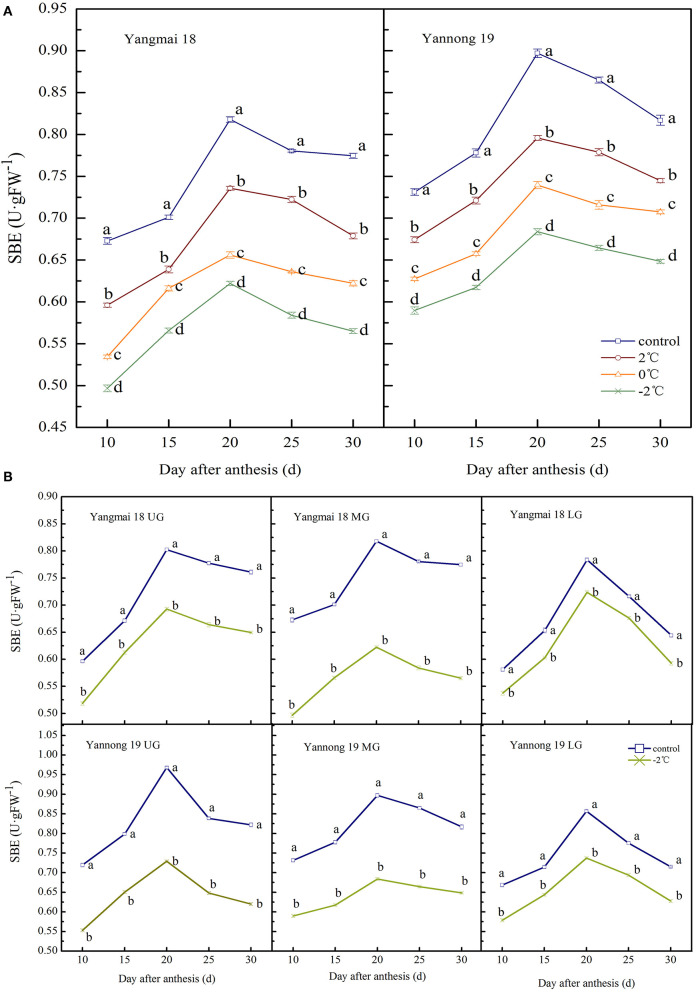
The effect of short-term LT at the booting stage on the starch branching enzyme activity of wheat grains in the middle spikelets **(A)** and at different position spikelets **(B)**. UG, grains in the upper spikelets; MG, grains in the middle spikelets; LG, grains in the lower spikelets. At the booting stage, wheat plants were placed in the artificial climate chamber for LT stress. The temperature was set to 5°C during 07:00–19:00 and 2, 0, and −2°C during 19:00–07:00. The normal growing plants without cold stress were used as controls. The data are presented as the means ± SD (*n* = 3). Different lowercase letters indicate significant differences between the treatments determined using the Duncan's multiple range test (*P* < 0.05).

The observed changes in the SBE activity of wheat grains during grain filling were similar to that of GBSS. The enzyme activity showed a single-peak curve that reached a peak around 20 days after anthesis (Figures 6A,B). After a short LT treatment at the booting stage, the SBE activity in the grains of the two varieties was significantly lower than the control group during the entire filling period (*P* < 0.05). At 20 days after anthesis, the SBE activity in the grains of the middle spikelet of the Yangmai 18 decreased by 10.03, 19.78, and 23.94% at corresponding temperatures of 2, 0, and −2°C, respectively. The Yannong 19 variety showed corresponding decreases of 11.28, 17.56, and 23.77%, respectively, compared to the controls.

The SBE activities in grains at different spike positions after treatment at −2°C was significantly lower than in the control group during the whole filling period (*P* < 0.05, Figure 6B). The peak SBE activity for grains in the upper, middle and lower spikelets of the Yangmai 18 grains decreased by 13.70, 23.94, and 7.66%, respectively. The corresponding SBE activity values in Yannong 19 grains decreased by 24.69, 23.77, and 13.96% compared to controls. Furthermore, the SBE activity of Yangmai 18 grains was lower than in the Yannong 19 variety during the whole filling period.

### The Effects of LT at the Booting Stage on the Content and Accumulation Rate of Starch in Grains

**Figure 7:**
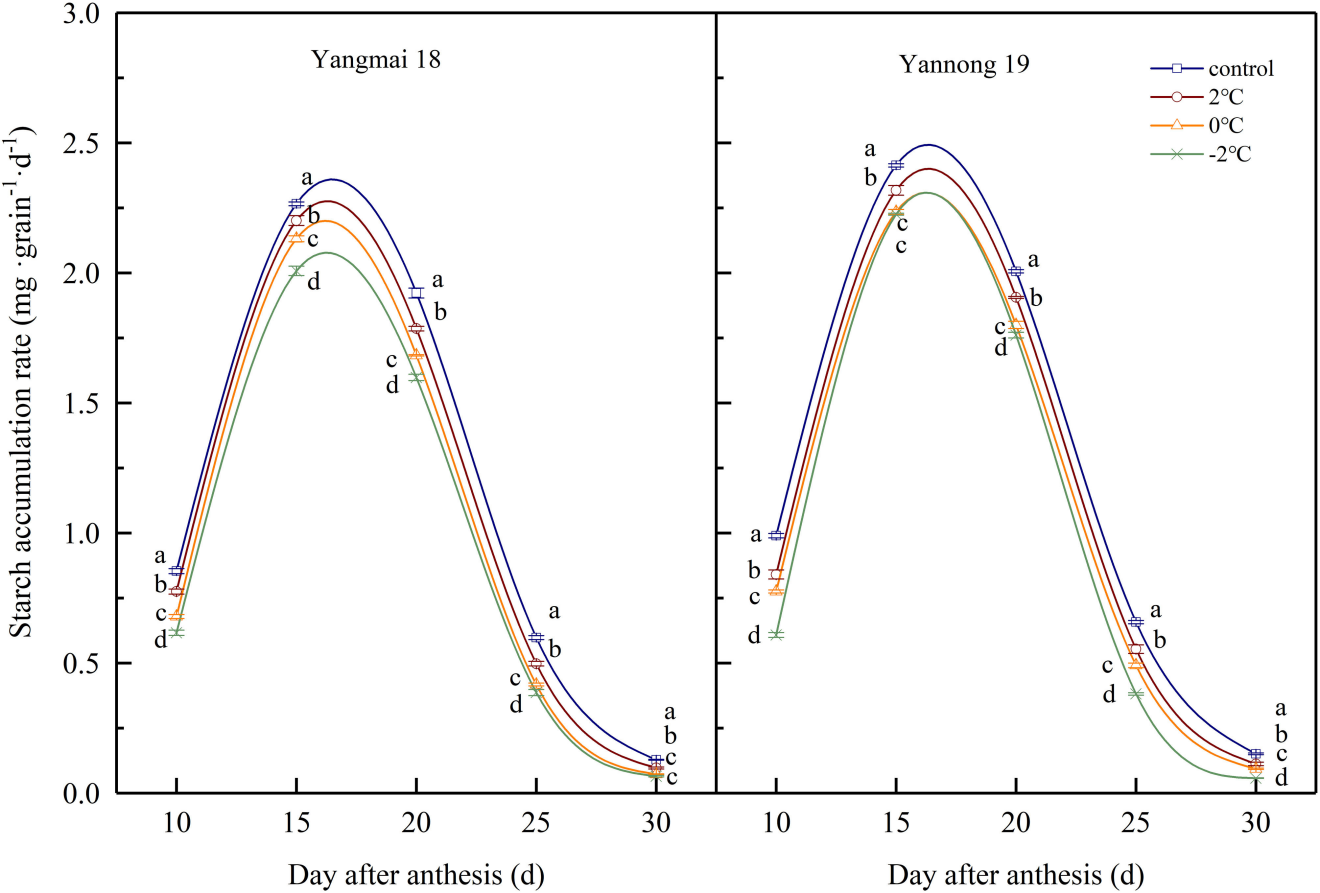
The effect of short-term LT at the booting stage on the rate of starch accumulation. At the booting stage, wheat plants were placed in an artificial climate chamber for LT stress. The temperature was set to 5°C during 07:00–19:00 and 2, 0, and −2°C during 19:00–07:00. The normal growing plants without cold stress were used as controls. The data are presented as the means ± SD (*n* = 3). Different lowercase letters indicate significant differences between the treatments determined using the Duncan's multiple range test (*P* < 0.05).

During wheat grain development, the starch contents gradually increased with time (Table 1). The starch contents increased rapidly at 15–20 d after anthesis and gradually stabilized at 25–30 d after anthesis. After short-term LT stress at the booting stage, the starch content of the grains was significantly lower compared to the control grains during the whole filling process (*P* < 0.05). These data indicated that LT stress at the booting stage reduced the accumulation of starch in wheat grains and lower temperatures had more significant impacts on starch accumulation. The rate of starch accumulation was obtained using logistic equations (Figure 7) and was maintained at a high level 10–20 days after anthesis before rapidly decreasing 25 days after anthesis. After LT stress at the booting stage, the rate of starch accumulation in grains was significantly lower than in control grains (*P* < 0.05). For example, the starch accumulation rate of grains of Yangmai 18 grains exposed to −2°C at the booting stage decreased by 17.69, 21.53, 6.55, 17.24, and 6.09% at days 10, 15, 20, 25, and 30 compared to control grains after anthesis.

**Table 1:** Effects of different treatments on starch content in wheat grains.

**Cultivar**	**Treatment**	**Days after anthesis (d)**				
		**10**	**15**	**20**	**25**	**30**
Yangmai 18	Control	7.03a ± 0.07	25.66a ± 0.33	55.99a ± 0.12	68.13a ± 0.32	72.67a ± 0.05
	2°C	6.20b ± 0.06	23.27b ± 0.34	50.87b ± 0.24	63.66b ± 0.18	68.27b ± 0.18
	0°C	4.86c ± 0.35	21.98c ± 0.06	47.52c ± 0.20	60.05c ± 0.30	65.10c ± 0.05
	−2°C	4.05d ± 0.09	20.30d ± 0.36	44.93d ± 0.33	55.83d ± 0.42	59.09d ± 0.20
Yannong 19	Control	8.94a ± 0.13	29.06a ± 0.14	59.69a ± 0.12	73.36a ± 0.32	77.49a ± 0.11
	2°C	7.07b ± 0.18	25.13b ± 0.22	54.77b ± 0.16	67.67b ± 0.22	72.28b ± 0.36
	0°C	6.17c ± 0.11	23.09c ± 0.12	51.96c ± 0.17	62.88c ± 0.08	69.58c ± 0.10
	−2°C	5.03d ± 0.26	20.39d ± 0.12	48.91d ± 0.12	59.52d ± 0.21	62.06d ± 0.11

*control: wheat plants grown without cold stress. 2, 0, and −2°C: wheat plants were placed in an artificial climate chamber during booting stage for 24 h. Temperature was set to 5°C during 07:00–19:00 and 2, 0, and −2°C during 19:00–07:00, respectively. Lowercase letters following the data refer to significant differences (P < 0.05)*.

### The Effects of LT at the Booting Stage on Dry Matter Accumulation in Wheat Grains

The logistic equation was used to fit the dynamics of the accumulation of grain dry matter (Table 2). The coefficient of determination (*R*^2^) of each fitting equation was highly significant (*P* < 0.01). The maximum theoretical thousand-grain weight of the wheat after short-term LT stress at the booting stage was lower than the control and the grain dry weight decreased concomitantly with the LT treatment. Exposure to short-term LT at the booting stage prolonged the period of incremental filling (*T*_1_) compared to the control and differentially affected the rapid filling (*T*_2_) and slow filling (*T*_3_) periods of the two varieties. Exposure to short-term LT at the booting stage prolonged the filling period (*T*), delayed the time of the maximum filling rate (*T*_max_) and reduced the mean filling rate (*R*) and the maximum filling rate (*R*_max_) (except for the −2°C treatment of Yangmai 18) compared to the control grains. These data indicated that the main reason for the observed decrease in the accumulation of dry matter in grains caused by short-term LT was a reduction in the rate of grain filling.

**Table 2:** Summary of the dry matter accumulation model and the parameters of wheat grain after short LT treatment during the booting stage.

**Cultivar**	**Treatment**	**Model**	**Decision coefficient (*R*^**2**^)**	***T*_**1**_ (d)**	***T*_**2**_ (d)**	***T*_**3**_ (d)**	***T*_**max**_ (d)**	***T* (d)**	***R* (g.1000grain^**−1**^.d^**−1**^)**	***R*_**max**_ (g.1000grain^**−1**^.d^**−1**^)**
Yangmai 18	Control	*Y* = 51.0948/[1 + e^(2.8665−0.162143**t**)^]	0.9969^**^	9.5564	16.2449	5.4287	17.6788	31.2300	1.6361	2.0712
	2°C	*Y* = 48.3310/[1 + e^(2.9566−0.168596**t**)^]	0.9986^**^	9.7250	15.6231	5.2209	17.5366	30.5691	1.5810	2.0371
	0°C	*Y* = 49.6990/[1 + e^(3.0789−0.160819**t**)^]	0.9997^**^	10.9558	16.3787	5.4734	19.1451	32.8078	1.5149	1.9981
	−2°C	*Y* = 46.6021/[1 + e^(3.3224−0.173897**t**)^]	0.9987^**^	11.5321	15.1469	5.0618	19.1056	31.7408	1.4682	2.0260
Yannong 19	Control	*Y* = 52.6116/[1 + e^(2.9721−0.163389**t**)^]	0.9986^**^	10.1298	16.1210	5.3873	18.1903	31.6381	1.6629	2.1490
	2°C	*Y* = 51.9728/[1 + e^(2.9072−0.147275**t**)^]	0.9935^**^	10.7975	17.8849	5.9767	19.7399	34.6591	1.4995	1.9136
	0°C	*Y* = 49.9081/[1 + e^(3.0761−0.154589**t**)^]	0.9989^**^	11.3792	17.0387	5.6940	19.8986	34.1119	1.4631	1.9288
	−2°C	*Y* = 48.5157/[1 + e^(2.9530−0.146178**t**)^]	0.9990^**^	11.1918	18.0191	6.0216	20.2014	35.2326	1.3770	1.7730

*Control: wheat plants grown without LT stress; 2, 0, and −2°C: wheat plants were placed in an artificial climate chamber during the booting stage for 24 h. The temperature was set to 5°C during 07:00–19:00 and 2, 0, and −2°C during 19:00–07:00*.

*Y: the weight of 1,000 grains. t: the day after anthesis. T_1_, T_2_, and T_3_: the duration of grain filling increasing period, the fast increasing period, and slow increasing period. T_max_: the time of maximum grain filling rate. T: the time of maximum dry matter mass in grain. R: average grain filling rate. R_max_: maximum grain filling rate*.

** *indicate significant differences at the 0.01 level*.

### The Effects of LT at the Booting Stage on the Number of Grains per Spike and Thousand-Grain Weight

**Figure 8:**
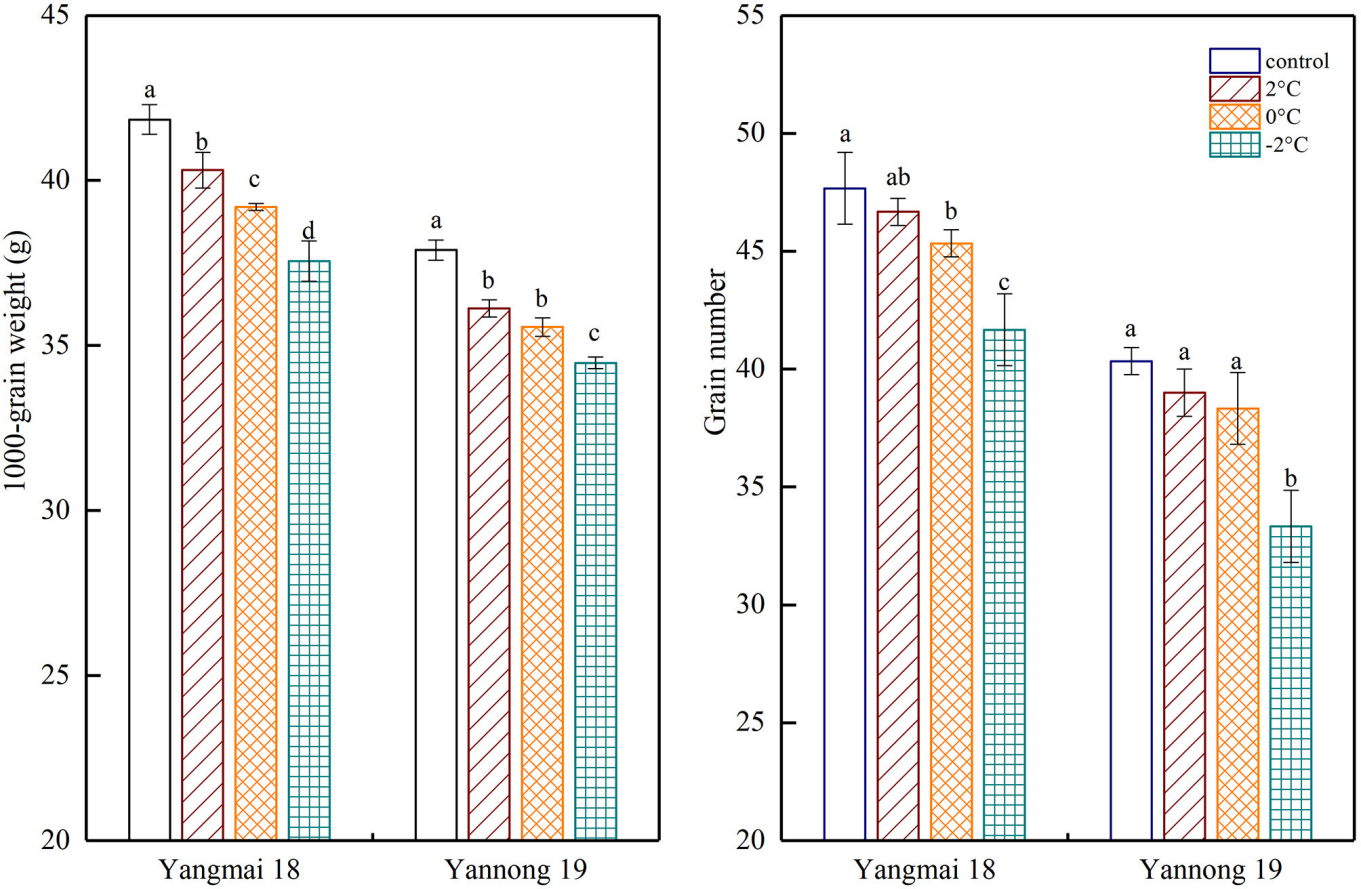
The effect of short-term LT at the booting stage on the 1000-grain weight and the grain number. At the booting stage, wheat plants were placed in an artificial climate chamber for LT stress. The temperature was set to 5°C during 07:00–19:00 and 2, 0, and −2°C during 19:00–07:00. The normal growing plants without cold stress were used as controls. The data are presented as the means ± SD (*n* = 3). Different lowercase letters indicate significant differences between the treatments determined using the Duncan's multiple range test (*P* < 0.05).

Exposure to short-term LT stress at the booting stage decreased the final number of grains per spike and the thousand-grain weight (Figure 8). A significant difference was observed between the thousand-grain weight of the Yangmai 18 variety at different temperatures (*P* < 0.05). However, no significant difference was observed in the number of grains per spike at 2°C compared to the control samples. Further temperature reductions to 0°C and −2°C led to significant differences in the thousand-grain weight compared to the control samples. In the Yannong 19 grains, a notable difference between LT treatments and the controls (*P* < 0.05), however, the only significant difference in the number of grains per spike was detected at a temperature of −2°C. A short period of LT at the booting stage greatly affected the thousand-grain weight and the impact was greater in the temperature-sensitive Yangmai 18 variety.

### Correlation Analysis of the Rate of Wheat Starch Accumulation, Starch Content, Thousand-Grain Weight, and the Activity of Enzymes Related to Starch Synthesis Under Short-Term LT Conditions at the Booting Stage

The relationships between starch accumulation rate, starch content, thousand-grain weight and the activities of enzymes related to starch synthesis were analyzed and the data presented in Table 3. After short-term LT stress at the booting stage, a positive correlation was observed between the rate of starch accumulation, starch content and the activities of AGPase and SSS. The activities of SSS and AGPase were very strongly correlated with the rate of starch accumulation (*P* < 0.05). The AGPase activity was also significantly correlated with the starch content in the Yangmai 18 grains. Also, the activities of GBSS and SBE were positively correlated with starch content and thousand-grain weight. The correlation between the SBE activity in the two different grain varieties and the starch content and the thousand-grain weight was highly significant. The correlation between GBSS activity, the starch content and the thousand-grain weight was highly significant for the Yannong 19, whereas the same correlation in the Yangmai 18 was not significant.

**Table 3:** Summary of the correlation coefficients between the rate of starch accumulation, the starch content, the 1000-grain weight, and the enzyme activities after LT stress at booting stage.

**Cultivars**		**AGPase**	**SSS**	**GBSS**	**SBE**
Yangmai 18	SAR	0.5539*	0.8531**	0.4395	0.1316
	SC	0.5176*	0.0993	0.3687	0.5696**
	GW	0.4089	−0.0232	0.2555	0.5079*
Yannong 19	SAR	0.5608*	0.7254**	0.1912	0.0921
	SC	0.4182	0.3175	0.7283**	0.5855**
	GW	0.2255	0.142	0.6080**	0.5129*

*AGPase, ADP-glucose pyrophosphorylase activity (U.g^−1^FW); SSS, soluble starch synthase activity (U.g^−1^FW); GBSS, granule-bound starch synthase activity (U.g^−1^FW); SBE, starch branching enzyme activity (U.g^−1^FW); SAR, starch accumulation rate (g.grain^−1^.d^−1^); SC, starch content (mg.g^−1^DW); GW, 1000-grain weight (g)*.

* and ** indicate significant differences at 0.05 and 0.01 level, R_0.05_ = 0.4438, R_0.01_ = 0.5614.

## Discussion

### The Effects of Short-Term LT at the Booting Stage on the Activities of Enzymes Related to Starch Synthesis

In the processes of starch synthesis, a variety of enzymes participate in the metabolism of carbohydrates during endosperm development in cereal (Hannah and James, 2008, Chen et al., 2020). The synthesis and accumulation of grain starch are affected by genetic factors and environmental conditions. The activities of key starch synthesis enzymes during grain development are extremely sensitive to ambient temperature that can directly affect the synthesis of starch in wheat grains (Thitisaksakul et al., 2012; Davinder et al., 2018). For example, high temperatures after anthesis can significantly reduce the activities of AGPase, SSS, GBSS, sucrose synthase (SS), and SBE in wheat grains (Lu et al., 2019) whilst also reducing the relative expression of genes encoding GBSS, SBE, and AGPase (Zhao et al., 2008).

LT stress significantly decreases the activities of SS, SSS, and AGPase during the corn filling stage. These changes cause the reduced conversion of starch synthesis substrates and starch accumulation efficiency (Zhang et al., 2018). We demonstrated that the activities of key starch synthesis enzymes (AGPase, SSS, GBSS and SBE) during the grain-filling stage all showed a single-peak curve. After LT stress at the booting stage, the activities of the four key starch synthesis enzymes decreased to varying degrees in grains at different spike positions. Previous studies reported that under high-temperature conditions, the peak activities of AGPase and SSS in rice grains precede those of GBSS and SBE (Jiang et al., 2003; Cheng et al., 2005).

In this study, a peak in enzyme activity was generally observed around 20 days after anthesis, except for AGPase activity in the upper and lower spike grains of Yannong 19 grains where the peak was delayed and occurred 25 days after anthesis. Luo et al. (in press) found that AGPase, SSS, and SBE activities in superior and inferior grains played key roles in regulating starch synthesis and were closely correlated with the asynchronism grain filling (Zhao et al., 2021). This suggests that LT may have different effects on the activity of AGPase in grains at different spike positions. In our study, the upper and lower grains in the Yannong 19 variety that differentiate and develop later were more affected by LT. These observations did not occur in the sensitive Yangmai 18 variety and require further investigation to determine whether its role in floret development.

SSS catalyzes the synthesis of amylopectin which accounts for 65–78% of starch content in grains (Zhang et al., 2010). It is acknowledged that high SSS activity represents a high ability to synthesize amylopectin from the substrate ADPG (Zhang G. Y. et al., 2011). In this study, the two wheat varieties suffered short-term LT stress at the booting stage and the SSS activities at different spike positions were not consistent. The SSS activity in the middle spikelet grains was generally low and differed slightly from the control at an early filling stage (10–15 d after anthesis). However, differences in SSS activity at the middle and late stages of filling (15–30 d after anthesis) increased and may be due to decreases in AGPase activity leading to decreases in the amount of ADPG substrate that effects the activity of SSS.

Kumar et al. (2017) observed decrease in the activity of SSS under terminal heat stress. The influence of temperature changes on the activity of SSS is the main reason for reductions in starch synthesis. Other studies suggest that LT in the early and middle stages of corn grain filling mainly regulates starch synthesis and grain filling by affecting the activity of SSS in the grain (Zhang et al., 2018). In this study, a short period of LT (−2°C) at the booting stage decreased SSS activity during the filling stage, however, this effect was not always lower than the control in the upper and lower spikelet grains during the entire filling period. These data suggest a spike position-specific effect of LT during the booting stage. These observations may be related to the asynchronous development of florets at different spike positions, however, further investigations are required to better understand the importance of these findings.

Under LT conditions, gibberellin suppresses potato starch synthesis by downregulating the expression of genes that encode AGPase and GBSS (Xie et al., 2018). In rice, high temperature could decrease GBSS activity in grains and significantly reduce amylose content (Ahmed et al., 2015). In this study, the activity of GBSS in grains at different spike positions after a short period of LT at the booting stage was significantly lower than in the control grains during the entire filling stage which affected the synthesis of starch in the grain, particularly the synthesis of amylose. SBE is the key enzyme involved in the synthesis of amylopectin which catalyzes the conversion of amylose to amylopectin (Abe et al., 2014; Brummell et al., 2015). At the mature grain stage, LT caused a decrease in the activities of SUSase and SBE in rice endosperm, affecting starch synthesis and delayed grain maturation (Baek et al., 2018).

In contrast to the duration of LT, the LT level had a greater impact on the nutritional quality of wheat grains. Also, the amylopectin content is more sensitive to LT compared to the amylose content (Liu et al., 2019). Our data support these previous findings. A short period of LT at the booting stage led to a significant decrease in the activity of SBE in grains at different spike positions. These changes were proportional to the decrease in temperature and were more prominent in the sensitive variety compared to the tolerant variety. This study showed that the short-term LT at the booting stage influenced the activities of key enzymes involved in starch synthesis. Temperature decreases directly leading to decreases in the activities of enzymes involved in starch synthesis. The lower the treatment temperature, the greater the enzyme activity decreased.

Furthermore, there is evidence that the plastidial starch phosphorylase (Pho1) and plastidial disproportionating enzyme (DpeI) are major players in starch biosynthesis under low temperatures (Hwang et al., 2019; Koper et al., 2021). Pho1 is essential for starch initiation and reduce the starch accumulation at low temperature (Satoh et al., 2008). Next, we can further investigate the mechanisms of Pho1 and DpeI affecting starch synthesis together with the above key enzymes after short-term LT at the booting.

### The Effects of Short-Term LT at the Booting Stage on the Accumulation of Starch Grains

The filling process of wheat grain mainly involves starch synthesis and accumulation. Stresses such as high temperatures and drought affect the activity of key starch synthesis enzymes in grains and impact grain development that in turn act to decreases the accumulation of starch grains (Asthir et al., 2012; He et al., 2012; Yi et al., 2014). Jenner et al. (1991) proposed that after anthesis high temperatures promote the accumulation of starch in the endosperm leading to a shorter filling period. High temperatures during the filling stage reduced the activity of starch synthase (Yang et al., 2018) and decreases metabolite levels in developing wheat grains (Asthir et al., 2012).

In this study, a short period of LT at the booting stage led to a lower starch content in wheat grains during the entire filling process which is consistent with previous results on the effect of high temperature after anthesis (Liu et al., 2017). This study also showed that the decrease in starch content was proportional to the decrease in temperature. This may be due to the inhibitory effect of LT at the booting stage on photosynthesis resulting in decreased carbon accumulation that reduces the synthesis and accumulation of starch and its components (Li et al., 2015).

Studies have also shown that LT during the maturation phase does not significantly affect total starch content but does affect the amylose content (Ahmed et al., 2015; Baek et al., 2018). Therefore, it is necessary to investigate whether exposure to short-term LT at the booting stage affects the starch composition of grains. Sreenivasulu et al. (2010) observed that the peak in starch accumulation generally occurs during the grain-filling stage, 12–35 days after anthesis. In this study, the rate of starch accumulation in the two wheat cultivars was maintained at a relatively high level 10–20 days after anthesis and then declined rapidly. These observations are consistent with the timing of the peak value and subsequent changes in the activity of starch synthase. After anthesis, the temperature of the canopy and the photosynthetic capacity decrease leading to decreases in the weight of single grains, the rate of starch synthesis, and starch accumulation (Savitch et al., 1997; Barnabás et al., 2008).

In this study, the rate of starch accumulation in the two wheat varieties after a short period of LT at the booting stage was lower than the control grains. This may be due to LT negatively affecting the developmental stage of young ears which consequently inhibits the synthesis of organic matter and changes the source-sink relationships (Zhang B. et al., 2015). These changes affect the supply of organic nutrients during endosperm cell proliferation and enrichment resulting in poor grain growth and a lower rate of starch synthesis during the later stages of development (Savitch et al., 1997). Cold stress after anthesis promotes the transfer of soluble proteins from leaves to grains and promotes the synthesis of grain proteins. It can also inhibit the rate of synthesis of grain starch to reduce starch content. Cold stress also inhibits grain lengths and volume leading to expanded and abnormal starch granules (Labuschagne et al., 2009; Wang et al., 2016).

### The Effects of Short-Term LT at the Booting Stage on Grain Dry-Matter Accumulation and Yield

LT affects wheat yield by reducing the accumulation of grain dry matter and the number of grains per spike (Valluru et al., 2012; Frederiks et al., 2015), and reduces the rate of grain filling (Huang et al., 2013). LT during grain filling in rice reduces the filling rate, delays the filling process and ultimately leads to the insufficient accumulation of grain dry-matter (Ahmed et al., 2008). Our data showed that exposure to short-term LT at the booting stage reduced the mean and maximum rates of grain-filling rate in wheat resulting in decreased dry-matter accumulation (Gómez and Zamora, 2003). Furthermore, LT at the booting stage prolonged the time of the incremental filling period and delayed the maximum filling rate as wheat plants are more sensitive to LT during this stage. Temperatures lower than 0°C seriously affect the absorption and distribution of nutrient elements and reduce the rate of grain filling resulting in lower grain quality (Fuller et al., 2007). Ahmed et al. (2008) also reported that LT delays the entire filling process by reducing the maximum filling rate. We also observed that LT stress at the booting stage significantly reduced the mean filling rate in the two grain varieties with the sensitive Yangmai 18 grain showing a more significant decrease compared to the tolerant Yannong 19 grain.

The exposure of wheat to LT in spring results in low wheat yield due to the decline of tillers and the production of fewer grains in spikelets. These changes reduce the number of spikes per area and the number of grains per spike (Li et al., 2015; Martino and Abbate, 2019). In this study, the final grain number and the thousand-grain weight after short-term LT stress at the booting stage decreased which is consistent with previous studies in rice (Zhu et al., 2017). Furthermore, the decrease in thousand-grain weight and grain number per ear was proportional to the decrease in temperature.

LT in spring cause assimilates in wheat to move from the stem to the leaves, leading to a decreased accumulation of dry matter and ultimately reducing the yield (Poorter et al., 2012). LT especially at booting also caused increased spikelet sterility and decreased grain yield (Osman et al., 2020) and LT at the booting stage affects spikelet sucrose metabolism and hormone content, resulting in a decrease in the final grain number and thousand-grain weight (Zhang W. J. et al., 2019). LT at the jointing and booting stages also significantly reduced the number of effective tillers per plant (Li et al., 2015) and can result in chill-induced pollen sterility affecting the growth of young ears (Frederiks et al., 2015). In the present study, LT at the booting stage had a greater impact on the thousand-grain weight than on the number of grains per spike, and affected the sensitive variety more. Previous reports showed that the LT at the jointing and booting stages had a greater impact on the number of spikes and grains per spike than on the number of grains per spike (Ji et al., 2017). This is because the LT level used in this experiment had little effect on floret development in the tolerant variety, and only the number of grains per spike at −2°C differed significantly from that of the control.

### The Relationship Between the Activities of Key Starch Synthesis Enzymes, Starch Synthesis, and the Accumulation of Dry Matter in Grains Following Short-Term LT at the Booting Stage

The AGPase and SS are the key regulatory enzymes involved in the starch biosynthesis, and the expression of its encoding gene is positively correlated with starch accumulation and content (Kittiwongwattana, 2019; Prathap and Tyagi, 2020). Starch accumulation is positively associated with AGPase activity in wheat endosperm (Verma et al., 2020). Zi et al. (2018) found that the activities of AGPase, SSS and GBSS in wheat endosperm were positively correlated with the contents of starch and amylopectin. Similar results were obtained in this study showing that changes in the activity of key enzymes involved in starch synthesis following short-term LT stress at the booting stage were closely related to the rate of starch accumulation, starch content and grain dry matter accumulation.

The observed changes in the activity of key enzymes involved in starch synthesis coincided with the rate of starch accumulation that reached a peak around 20 days after anthesis. The activities of AGPase and SSS were significantly positively correlated with the rate of starch accumulation and SSS activity was significantly positively correlated with the rate of starch accumulation. Studies have shown that the rate of rice grain filling is significantly related to the activity of SBE (Baek et al., 2018). In this study, the activities of GBSS and SBE were weakly correlated with the rate of starch accumulation but positively correlated with changes in starch content and the thousand-grain weight. In particular, the correlation between SBE activity, starch content and the thousand-grain weight was significant. From these data, it can be inferred that LT at the booting stage affects the development of spikelets and florets to reduce the production and supply of starch synthesis substrates in the grain. At the same time, LT significantly reduces the activity of key starch synthesis enzymes, reduces the conversion of starch synthesis substrates and the efficiency of starch accumulation. Collectively, these changes result in a decreased rate of starch synthesis leading to lower starch content and dry matter weight in the mature grain.

## Conclusion

Exposure to short-term LT at the booting stage has a major impact on the starch synthesis and dry-matter accumulation in wheat grains during grain filling. The activities of key starch synthesis enzymes (AGPase, SSS, GBSS, and SBE) decreased to varying degrees throughout the filling period, whilst the rate of starch accumulation and the starch content gradually decreased with decreases in treatment temperature. The accumulation period of grain dry matter was prolonged but the accumulation rate decreased, and the timing of the maximum accumulation rate was delayed. The number of grains per spike and the thousand-grain weight also decreased.

Our data show that in wheat grains, starch synthesis and grain filling are subject to complex regulatory mechanisms during the booting stage. The influence of LT on other key enzymes for starch synthesis and starch components requires further investigation.

## Data Availability Statement

The raw data supporting the conclusions of this article will be made available by the authors, without undue reservation.

## Author Contributions

WZ and ZH designed the experiment. WZ conducted the study and wrote the manuscript. YZ, LL, XX, LY, ZL, and BW performed the experiments and helped in sampling and the physiological parameter measurements. SM and YF helped in drafting the manuscript. All authors read and approved the final manuscript.

## Conflict of Interest

The authors declare that the research was conducted in the absence of any commercial or financial relationships that could be construed as a potential conflict of interest.

## References

[B1] Abe N. Asai H. Yago H. Oitome N. F. Itoh R. Crofts N.. (2014). Relationships between starch synthase i and branching enzyme isozymes determined using double mutant rice lines. BMC Plant Biol. 14:80. 10.1186/1471-2229-14-80 24670252 PMC3976638

[B2] Ahmed N. Maekawa M. Tetlow I. J. (2008). Effects of low temperature on grain filling, amylose content, and activity of starch biosynthesis enzymes in endosperm of basmati rice. Aust. J. Agr. Res. 59, 599–604. 10.1071/AR07340 25284759

[B3] Ahmed N. Tetlow I. J. Nawaz S. Iqbal A. Mubin M. Rehman M. S. N. U.. (2015). Effect of high temperature on grain filling period, yield, amylose content, and activity of starch biosynthesis enzymes in endosperm of basmati rice. J. Sci. Food Agr. 95, 2237–2243. 10.1002/jsfa.6941 25284759

[B4] Asthir B. Rai P. K. Bains N. S. Sohu V. S. (2012). Genotypic variation for high temperature tolerance in relation to carbon partitioning and grain sink activity in wheat. Am. J. Plant Sci. 3, 381–390. 10.4236/ajps.2012.33046

[B5] Baek J. S. Jeong H. Y. An S. H. Jeong J. H. Lee H. S. Yoon J. T.. (2018). Effects of low temperature during ripening on amylose content and enzyme activities associated with starch biosynthesis in rice endosperm. Korean J. Crop Sci. 63, 86–97. 10.7740/kjcs.2018.63.2.086

[B6] Barnabás B. Jäger K. Fehér A. (2008). The effect of drought and heat stress on reproductive reproductive processes in cereals. Plant Cell Environ. 31, 11–38. 10.1111/j.1365-3040.2007.01727.x 17971069

[B7] Bowsher C. G. Scrase-Field E. F. A. L. Esposito S. Emes M. J. Tetlow I. J. (2007). Characterization of ADP-glucose transport across the cereal endosperm amyloplast envelope. J. Exp. Bot. 58, 1321–1332. 10.1093/jxb/erl297 17301030

[B8] Brummell D. A. Watson L. M. Zhou J. McKenzie M. J. Hallett I. C. Simmons L.. (2015). Overexpression of starch branching enzyme II increases short-chain branching of amylopectin and alters the physicochemical properties of starch from potato tuber. BMC Biotechnol. 15, 1–14. 10.1186/s12896-015-0143-y 25926043 PMC4414359

[B9] Cao Z. Z. Pan G. Wang F. B. Wei K. S. Li Z. W. Shi C. H.. (2015). Effect of high temperature on the expressions of genes encoding starch synthesis enzymes in developing rice endosperms. J. Integr. Agr. 14, 642–659. 10.1016/S2095-3119(14)60782-6

[B10] Chen Y. L. Pang Y. H. Bao J. S. (2020). Expression profiles and protein complexes of starch biosynthetic enzymes from white-core and waxy mutants induced from high amylose indica rice. Rice Sci. 27, 152–161. 10.1016/j.rsci.2020.01.006

[B11] Cheng F. M. Zhong L. J. Zhao N. C. Liu Y. Zhang G. P. (2005). Temperature induced changes in the starch components and biosynthetic enzymes of two rice varieties. Plant Growth Regul. 46, 87–95. 10.1007/s10725-005-7361-6

[B12] Crimp S. J. Zheng B. Y. Khimashia N. Gobbett D. L. Chapman S. Howden M. (2016). Recent changes in southern Australian frost occurrence: implications for wheat production risk. Crop Pasture Sci. 67, 801–811. 10.1071/CP16056

[B13] Darroch B. A. Baker R. J. (1990). Grain filling in three spring wheat genotype: statistical analysis. Crop Sci. 30, 525–529. 10.2135/cropsci1990.0011183X003000030009x

[B14] Davinder S. Ratan T. Vijay K. G. Jagadish R. Rajender S. (2018). Genotype and ambient temperature during growth can determine the quality of starch from wheat. J. Cereal Sci. 79, 240–246. 10.1016/j.jcs.2017.11.006

[B15] Fang S. B. Yang J. J. Zhou G. S. (2011). Change trend distributive characteristics of agrometeorological disasters in China in recent 30 years. J. Nat. Dis. 20, 69–73. 10.13577/j.jnd.2011.0511

[B16] Frederiks T. M. Christopher J. T. Sutherland M. W. Borrell A. K. (2015). Post-head-emergence frost in wheat and barley: defining the problem, assessing the damage, and identifying resistance. J. Exp. Bot. 66, 3487–3498. 10.1093/jxb/erv088 25873656

[B17] Fuller M. P. Fuller A. M. Kaniouras S. Christophers J. Fredericks T. (2007). The freezing characteristics of wheat at ear emergence. Eur. J. Agron. 26, 435–441. 10.1016/j.eja.2007.01.001

[B18] Gómez J. M. Zamora R. (2003). Factors affecting intrafruit pattern of ovule abortion and seed production in Hormathophylla spinosa. Plant Syst. Evol. 239, 215–229. 10.1007/s00606-003-0009-y

[B19] Hannah L. C. James M. (2008). The complexities of starch biosynthesis in cereal endosperms. Curr. Opin. Biotechnol. 19, 160–165. 10.1016/j.copbio.2008.02.013 18400487

[B20] He J. F. Goyal R. Laroche A. Zhao M. L. Lu Z. X. (2012). Water stress during grain development affects starch synthesis, composition and physicochemical properties in triticale. J. Cereal Sci. 56, 552–560. 10.1016/j.jcs.2012.07.011

[B21] He W. Wei C. X. (2020). A critical review on structural properties and formation mechanism of heterogeneous starch granules in cereal endosperm lacking starch branching enzyme. Food Hydrocolloid. 100:105434. 10.1016/j.foodhyd.2019.105434

[B22] Holman J. D. Schlegel A. J. Thompson C. R. Lingenfelser J. E. (2011). Influence of precipitation, temperature, and 56 years on winter wheat yields in western Kansas. Crop Manag. 10, 1–10. 10.1094/CM-2011-1229-01-RS

[B23] Huang M. Jiang L. Zou Y. Zhang W. (2013). On-farm assessment of effect of low temperature at seedling stage on early-season rice quality. Field Crop. Res. 141, 63–68. 10.1016/j.fcr.2012.10.019

[B24] Hurkman W. J. McCue K. F. Altenbach S. B. Korn A. Tanaka C. K. Kothari K. M.. (2003). Effect of temperature on expression of genes encoding enzymes for starch biosynthesis in developing wheat endosperm. Plant Sci. 164, 873–881. 10.1016/S0168-9452(03)00076-1

[B25] Hwang S. K. Koper K. Okita T. W. (2019). The plastid phosphorylase as a multiple-role player in plant metabolism. Plant Sci. 290:110303. 10.1016/j.plantsci.2019.110303 31779913

[B26] Jenner C. F. Ugalde T. D. Aspinall D. (1991). The physiology of starch and protein desposition in the endosperm of wheat. Aust. J. Plant Physiol. 18, 211–226. 10.1071/PP9910211

[B27] Ji H. T. Xiao L. J. Xia Y. M. Song H. Liu B. Tang L.. (2017). Effects of jointing and booting low temperature stresses on grain yield and yield components in wheat. Agr. Forest Meteorol. 243, 33–42. 10.1016/j.agrformet.2017.04.016

[B28] Jiang D. Cao W. X. Dai T. B. Jing Q. (2003). Activities of key enzymes for starch synthesis in relation to growth of superior and inferior grains on winter wheat (*Triticum aestivum* L.) spike. Plant Growth Regul. 41, 247–257. 10.1023/B:GROW.0000007500.90240.7d

[B29] Kittiwongwattana C. (2019). Differential effects of synthetic media on long-term growth, starch accumulation and transcription of ADP-glucosepyrophosphorylase subunit genes in Landoltia punctata. Sci. Rep. 9, 971–985. 10.1038/s41598-019-51677-w 31653895 PMC6814796

[B30] Koper K. Hwang S. K. Wood M. Singh S. Cousins A. Kirchhoff H.. (2021). The rice plastidial phosphorylase participates directly in both sink and source processes. Plant Cell Physiol. 62, 125–142. 10.1093/pcp/pcaa146 33237266

[B31] Kumar R. R. Suneha G. Mohammed S. Upama M. Monika J. Khushboo S.. (2017). Biochemical defense response: characterizing the plasticity of source and sink in spring wheat under terminal heat stress. Front. Plant Sci. 8:1603. 10.3389/fpls.2017.01603 28979274 PMC5611565

[B32] Labuschagne M. T. Elago O. Koen E. (2009). The influence of temperature extremes on some quality and starch characteristics in bread, biscuit and durum wheat. J. Cereal Sci. 49, 184–189. 10.1016/j.jcs.2008.09.001

[B33] Li X. N. Pu H. C. Liu F. L. Zhou Q. Cai J. Dai T. B.. (2015). Winter wheat photosynthesis and grain yield responses to spring freeze. Agron. J. 107, 1002–1010. 10.2134/agronj14.0460

[B34] Liu L. L. Ma J. F. Tian L. Y. Wang S. H. Tang L. Cao W. X.. (2017). Effects of postanthesis high temperature on grain quality formation for wheat. Agron. J. 109, 1970–1980. 10.2134/agronj2016.07.0427

[B35] Liu L. L. Song H. Shi K. J. Liu B. Zhang Y. Tang L.. (2019). Response of wheat grain quality to low temperature during jointing and booting stages—on the importance of considering canopy temperature. Agr. Forest Meteorol. 278:107658. 10.1016/j.agrformet.2019.107658

[B36] Lu H. F. Hu Y. Y. Wang C. Y. Liu W. X. Ma G. Han Q. X.. (2019). Effects of high temperature and drought stress on the expression of gene encoding enzymes and the activity of key enzymes involved in starch biosynthesis in wheat grains. Front. Plant Sci. 10:1414. 10.3389/fpls.2019.01414 31798603 PMC6863091

[B37] Luo J. Li Z. Mo F. Liao Y. C. Liu Y. (in press). Removal of superior wheat kernels promotes filling of inferior kernels by changing carbohydrate metabolism and sink strength. The Crop J. 10.1016/j.cj.2020.12.012

[B38] Mahla R. Madan S. Kaur V. Munjal R. Midathala R. Midathala R. (2017). Activities of sucrose to starch metabolizing enzymes during grain filling in late sown wheat under water stress. J. Appl. Nat. Sci. 9, 338–343. 10.31018/jans.v9i1.1193

[B39] Martino D. L. Abbate P. E. (2019). Frost damage on grain number in wheat at different spike developmental stages and its modelling. Eur. J. Agron. 103, 13–23. 10.1016/j.eja.2018.10.010

[B40] Nakamura Y. Yuki K. Park S. Y. Ohya T. (1989). Carbohydrate metabolism in the developing endosperm of rice grains. Plant Cell Physiol. 30, 833–839. 10.1093/oxfordjournals.pcp.a077813

[B41] Osman R. Zhu Y. Ma W. Zhang D. Z. Ding Z. F. Liu L. L.. (2020). Comparison of wheat simulation models for impacts of extreme temperature stress on grain quality. Agr. Forest Meteorol. 15, 288–289. 10.1016/j.agrformet.2020.107995

[B42] Poorter H. Niklas K. J. Reich P. B. Oleksyn J. Poot P. Mommer L. (2012). Biomass allocation to leaves, stems and roots: meta-analyses of interspecific variation and environmental control. New Phytol. 193, 30–50. 10.1111/j.1469-8137.2011.03952.x 22085245

[B43] Prathap V. Tyagi A. (2020). Correlation between expression and activity of ADP glucose pyrophosphorylase and starch synthase and their role in starch accumulation during grain filling under drought stress in rice. Plant Physiol. Bioch. 157, 239–243. 10.1016/j.plaphy.2020.10.018 33130401

[B44] Ran L. P. Yu X. R. Li Y. Q. Zou J. C. Deng J. W. Pan J. Y.. (2020). Analysis of development, accumulation and structural characteristics of starch granule in wheat grain under nitrogen application. Int. J. Biol. Macromol. 164, 3739–3750. 10.1016/j.ijbiomac.2020.08.192 32871126

[B45] Satoh H. Shibahara K. Tokunaga T. Nishi A. Tasaki M. Hwang S. K.. (2008). Mutation of the plastidial glucan phosphorylase gene in rice affects the synthesis and structure of starch in the endosperm. Plant Cell 20, 1833–1849. 10.1105/tpc.107.054007 18621947 PMC2518224

[B46] Savitch L. V. Gray G. R. Huner N. P. A. (1997). Feedback-limited photosynthesis and regulation of sucrose-starch accumulation during cold acclimation and low-temperature stress in a spring and winter wheat. Planta 201, 18–26. 10.1007/BF01258676

[B47] Sreenivasulu N. Borisjuk L. Junker B. H. Mock H. P. Rolletschek H. Seiffert U.. (2010). Barley grain development toward an integrative view. Int. Rev. Cell Mol. Biol. 281, 49–89. 10.1016/S1937-6448(10)81002-0 20460183

[B48] Thitisaksakul M. Jiménez R. C. Arias M. C. Beckles D. M. (2012). Effects of environmental factors on cereal starch biosynthesis and composition. J. Cereal Sci. 56, 67–80. 10.1016/j.jcs.2012.04.002

[B49] Valluru R. Link J. Claupein W. (2012). Consequences of early chilling stress in two Triticum species: plastic responses and adaptive significance. Plant Biol. 14, 641–651. 10.1111/j.1438-8677.2011.00540.x 22309058

[B50] Verma V. C. Agrawal S. Kumar A. Jaiswal J. P. (2020). Starch content and activities of starch biosynthetic enzymes in wheat, rice and millets. J. Pharmacogn. Phytochem. 9, 1211–1218. 10.22271/phyto.2020.v9.i4q.11883

[B51] Wang S. Q. Song X. H. Zhao H. H. Sun M. M. Xiao C. L. Gu C. M.. (2016). Effect of chilling stress at booting stage on rice yield and quality in the chilling region. Res. Agr. Mod. 37, 579–586. 10.13872/j.1000-0275

[B52] Wang W. T. Cui W. P. Xu K. Gao H. Wei H. Y. Zhang H. C. (2021). Effects of early- and late-sowing on starch accumulation and associated enzyme activities during grain filling stage in rice. Rice Sci. 28, 191–199. 10.1016/j.rsci.2021.01.008

[B53] Wang Z. B. Li W. H. Qi J. C Shi P. C. Yin Y. A. (2014). Starch accumulation, activities of key enzyme and gene expression in starch synthesis of wheat endosperm with different starch contents. J. Food Sci. Technol. 51, 419–429. 10.1007/s13197-011-0520-z 24587516 PMC3931879

[B54] Xiao L. J. Liu B. Zhang H. X. Gu J. Y. Fu T. Y. Asseng S.. (2021). Modeling the response of winter wheat phenology to low temperature stress at elongation and booting stages. Agr. Forest Meteorol. 303:108376. 10.1016/j.agrformet.2021.108376

[B55] Xie Y. J. Onik J. C. Hu X. J. Duan Y. Q. Lin Q. (2018). Effects of (S)-carvone and gibberellin on sugar accumulation in potatoes during low temperature storage. Molecules 23:3118. 10.3390/molecules23123118 30487439 PMC6321255

[B56] Yang H. Gu X. T. Ding M. Q. Lu W. P. Lu D. L. (2018). Heat stress during grain filling affects activities of enzymes involved in grain protein and starch synthesis in waxy maize. Sci. Rep. 8, 1145–1156. 10.1038/s41598-018-33644-z 30353095 PMC6199321

[B57] Yang J. C. Zhang J. H. Wang Z. Q. Xu G. W. Zhu Q. S. (2004). Activities of key enzymes in sucrose-to-starch conversion in wheat grans subjected to water deficit during grain filling. Plant Physiol. 135, 1621–1629. 10.1104/pp.104.041038 15235118 PMC519076

[B58] Yi B. Zhou Y. F. Gao M. Y. Zhang Z. Han Y. Yang G. D.. (2014). Effect of drought stress during flowering stage on starch accumulation and starch synthesis enzymes in sorghum grains. J. Integr. Agr. 13, 2399–2406. 10.1016/S2095-3119(13)60694-2

[B59] Zeng Y. Yu J. Cang J. Liu L. J. Mu Y. C. Wang J. H.. (2011). Detection of sugar accumulation and expression levels of correlative key enzymes in winter wheat (*Triticum aestivum*) at low temperatures. Biosci. Biotechnol. Biochem. 75, 681–687. 10.1271/bbb.100813 21512254

[B60] Zhang B. Jia D. Gao Z. Q. Dong Q. He L. H. (2015). Physiological responses to low temperature in spring and winter wheat varieties. J. Sci. Food Agr. 96, 1967–1973. 10.1002/jsfa.7306 26095741

[B61] Zhang C. H. Jiang D. Liu F. L. Cai J. Dai T. B. Cao W. X. (2010). Starch granules size distribution in superior and inferior grain of wheat is related to enzyme activities and their gene expressions during grain filling. J. Cereal Sci. 51, 226–233. 10.1016/j.jcs.2009.12.002

[B62] Zhang G. Y. Cheng Z. J. Zhang X. Guo X. P. Su N. Jiang L.. (2011). Double repression of soluble starch synthase genes SSIIa and SSIIIa in rice (*Oryza sativa* L.) uncovers interactive effects on the physicochemical properties of starch. Genome 54, 448–459. 10.1139/g11-010 21595523

[B63] Zhang W. J. Huang Z. L. Wang Q. Guan Y. N. (2015). Effects of low temperature on leaf anatomy and photosynthetic performance in different genotypes of wheat following a rice crop. Int. J. Agric. Biol. 17, 1165–1171. 10.17957/IJAB/15.0035

[B64] Zhang W. J. Wang J. Q. Huang Z. L. Mi L. Xu K. F. Wu J. J.. (2019). Effects of low temperature at booting stage on sucrose metabolism and endogenous hormone contents in winter wheat spikelet. Front. Plant Sci. 10:498. 10.3389/fpls.2019.00498 31057594 PMC6482243

[B65] Zhang X. Hao J. P. Wang P. Zhang P. Chen L. J. (2018). Effects of low temperature on maize superior and inferior kernels development during grain filling *in vitro*. Scientia Agr. Sinica 51, 2263–2273. 10.3864/j.issn.0578-1752.2018.12.004

[B66] Zhang X. F. Zheng Y. F. Wang C. Y. Chen H. L. Ren Z. H. Zou C. H. (2011). Spatial distribution and temporal variation of the winter wheat late frost disaster in Henan, China. Acta Meteorol. Sin. 25, 249–259. 10.1007/s13351-011-0031-x

[B67] Zhao H. Dai T. Jiang D. Cao W. (2008). Effects of high temperature on key enzymes involved in starch and protein formation in grains of two wheat cultivars. J. Agron. Crop Sci. 194, 47–54. 10.1111/j.1439-037X.2007.00283.x

[B68] Zhao H. Li Z. Amjad H. Zhong G. P. Khan M. U. Zhang Z. X.. (2021). Proteomic analysis reveals a role of ADP-glucose pyrophosphorylase in the asynchronous filling of rice superior and inferior spikelets. Protein Expr. Purif. 183:105875. 10.1016/j.pep.2021.105875 33741528

[B69] Zheng B. Y. Chapman S. C. Christopher J. T. Frederiks T. M. Chenu K. (2015). Frost trends and their estimated impact on yield in the Australian wheatbelt. J. Exp. Bot. 66, 3611–3623. 10.1093/jxb/erv163 25922479 PMC4463805

[B70] Zhu D. W. Wei H. Y. Guo B. W. Dai Q. G. Wei C. X. Gao H.. (2017). The effects of chilling stress after anthesis on the physicochemical properties of rice (*Oryza sativa* L) starch. Food Chem. 237, 936–941. 10.1016/j.foodchem.2017.06.039 28764089

[B71] Zi Y. Ding J. F. Song J. M. Gavin H. Peng Y. X. Li C. Y.. (2018). Grain yield, starch content and activities of key enzymes of waxy and non-waxy wheat (*Triticum aestivum* L.). Sci. Rep. 8:4548. 10.1038/s41598-018-22587-0 29540822 PMC5852034

